# NCCAM/NCI Phase 1 Study of Mistletoe Extract and Gemcitabine in Patients with Advanced Solid Tumors

**DOI:** 10.1155/2013/964592

**Published:** 2013-10-27

**Authors:** Patrick J. Mansky, Dawn B. Wallerstedt, Timothy S. Sannes, Jamie Stagl, Laura Lee Johnson, Marc R. Blackman, Jean L. Grem, Sandra M. Swain, Brian P. Monahan

**Affiliations:** ^1^The Cancer Team at Bellin Health, 1580 Commanche Avenue, Green Bay, WI 54313, USA; ^2^National Center for Complementary and Alternative Medicine, NIH, Bethesda, MD, USA; ^3^Samuelli Institute, Alexandria, VA, USA; ^4^Department of Clinical and Health Psychology, University of Florida, Gainesville, FL, USA; ^5^University of Miami, Miami, FL, USA; ^6^Research Service (151), Veterans Affairs Medical Center, and Department of Medicine, Georgetown University School of Medicine, Washington, DC, USA; ^7^University of Nebraska Medical Center, Omaha, NE, USA; ^8^Washington Cancer Institute, Washington Hospital Center, Washington, DC, USA; ^9^Department of Medicine, Hematology and Medical Oncology Division, Uniformed Services University of the Health Sciences, Bethesda, MD, USA

## Abstract

*Purpose.* European Mistletoe (*Viscum album* L.) extracts (mistletoe) are commonly used for cancer treatment in Europe. This phase I study of gemcitabine (GEM) and mistletoe in advanced solid cancers (ASC) evaluated: (1) safety, toxicity, and maximum tolerated dose (MTD), (2) absolute neutrophil count (ANC) recovery, (3) formation of mistletoe lectin antibodies (ML ab), (4) cytokine plasma concentrations, (5) clinical response, and (6) pharmacokinetics of GEM. *Methods.* Design: increasing mistletoe and fixed GEM dose in stage I and increasing doses of GEM with a fixed dose of mistletoe in stage II. Dose limiting toxicities (DLT) were grade (G) 3 nonhematologic and G4 hematologic events; MTD was reached with 2 DLTs in one dosage level. Response in stage IV ASC was assessed with descriptive statistics. Statistical analyses examined clinical response/survival and ANC recovery. *Results.* DLTs were G4 neutropenia, G4 thrombocytopenia, G4 acute renal failure, and G3 cellulitis, attributed to mistletoe. GEM 1380 mg/m^2^ and mistletoe 250 mg combined were the MTD. Of 44 patients, 24 developed nonneutropenic fever and flu-like syndrome. GEM pharmacokinetics were unaffected by mistletoe. All patients developed ML3 IgG antibodies. ANC showed a trend to increase between baseline and cycle 2 in stage I dose escalation. 6% of patients showed partial response, 42% stable disease. Median survival was 200 days. Compliance with mistletoe injections was high. *Conclusion.* GEM plus mistletoe is well tolerated. No botanical/drug interactions were observed. Clinical response is similar to GEM alone.

## 1. Introduction

European mistletoe (*Viscum album* L.), a  semiparasitic plant growing on various trees [1], has been used in folklore and as a medicinal plant for several thousand years. In the modern era, it was first introduced as a plant extract preparation for the treatment of malignant diseases by Steiner [2]. A number of studies have reported immunostimulatory effects of mistletoe extracts, on mononuclear cells [3], lymphocytes [4–6], macrophages [7], and NK cells [8, 9]. Mistletoe extracts contain a number of biologically active components, including mistletoe lectins (reviewed in [10–16]) and viscotoxins [17, 18]. Mistletoe extracts may also have antiangiogenic properties [1]. Mistletoe lectins stimulate secretion of a number of cytokines including IL-6, IL-12, IL-1, and TNF-*α* [19–21], may enhance cytotoxic NK-cell activity, and may induced apoptosis [22] and induction of FAS ligand [23]. Some of these findings have been supported by microarray gene expression profiling [24]. Mistletoe extract reduces leukopenia in chemotherapy-treated mice and stimulates neutropoiesis in mice after cyclophosphamide chemotherapy [25]. In a dose-dependent fashion, ML-1 may upregulate protein synthesis in neutrophils at low doses, while high doses resulted in neutrophil apoptosis via a caspase-dependent mechanism [26]. Mixed findings have been reported on mistletoe antibody formation. *In vivo* antibody formation has a protective effect against the toxicity of mistletoe lectins to normal somatic cells [27]. While ML antibodies were absent in patients without adverse effects [28], a potential role of ML-antibodies in the neutralization of mistletoe lectin activity *in vivo* has been debated [27, 29]. Collectively, the mounting preclinical data with mistletoe therapy suggests that rigorous clinical trials are needed.

In a phase I study in HIV-positive patients treated with* Viscum album *Quercus Frischsaft (QuFrF) [30], limited toxicities included flu-like symptoms, gingivitis, eosinophilia, and a slight rise in serum urea nitrogen and creatinine. Natural mistletoe lectins were detected in normal volunteers 2 weeks after single dose injection. Fever and flu-like symptoms were observed [31]. Thus, mistletoe preparations appear to be well tolerated, and antibody response appears to be robust. However, the clinical efficacy of mistletoe in oncology settings remains unclear. Notably, in a large retrospective study of mistletoe therapy in nonmetastatic breast cancer patients, fewer adverse events and longer survival were observed in the mistletoe therapy group compared to conventional therapy alone [32]. In a comparable retrospective study design in pancreatic cancer patients, similar clinical outcomes were observed with fewer adverse events and improved survival in patients treated with mistletoe [33]. Despite compelling preclinical data and these isolated reports of clinical benefit of mistletoe preparations, recent reviews of clinical trials still note methodological weaknesses of current published studies on mistletoe [34] as well as conflicting results on tumor response and survival prolongation on treatment using various mistletoe preparations [35–38]. A number of recently published studies confirm this mixed picture [39–42].

With converging preclinical evidence suggesting immunostimulatory and antiangiogenic properties of mistletoe, in addition to a dearth of well-designed clinical trials testing the safety and efficacy of mistletoe, the present study sought to administer mistletoe to advanced stage cancer patients, in combination with a standard, well-known chemotherapy regimen (GEM), hypothesizing that gemcitabine and mistletoe can be administered safely in combination to patients with advanced cancer. The study aims were to evaluate the following: (1) safety, toxicity, and maximum tolerated dose (MTD) of the mistletoe/GEM combination in patients with advanced solid cancers (ASC), (2) neutrophil count recovery, (3) formation of mistletoe lectin antibodies (ML ab), (4) cytokine plasma concentrations, (5) clinical response, and (6) GEM pharmacokinetics as an indicator of possible interactions of the mistletoe/GEM combination regimen.

## 2. Materials and Methods

### 2.1. Mistletoe Extract Quality and Content Verification

A whole plant mistletoe extract (HELIXOR Apis (A), growing on fir trees), Lot 021224 and Lot 0406, was used and supplied by Helixor, GmbH, Rosenfeld, Germany. Study agent content analyses was performed by the manufacturer. Product content verification analyses were conducted by Lawrence Livermore National Laboratories, Livermore, CA, USA. Verification analyses were consistent with the manufacturer's analyses and showed no evidence for product contamination with pesticides, heavy metals, or the prescription drugs listed. The Helixor mistletoe extract was assayed for approximately 60 elemental species by inductively-coupled-plasma/mass spectrometry (ICP-MS). In addition, Helixor A mistletoe extract was assayed for a variety of pesticides and street drugs including stimulants, narcotics, and tranquilizers using gas chromatography-mass spectroscopy GC-MS.

Liquid Chromatography-Mass Spectrometry (LC-MS and LC-MS/MS) analysis of Helixor mistletoe formulation for commonly used oncology drugs was negative. Helixor A mistletoe extract was assayed by thin-layer chromatography (TLC) via a QA/QC protocol supplied by Helixor. TLC assay results of the submitted Helixor solution were very similar to those expected from the Helixor QA/QC protocol.

Both lots were tested in parallel up to this final assay performed on 14 February 2006. Lot 021224 contained 3.4 (±0.2) ng/mL ML-I and 178 (±4) ng/mL ML-III. Lot 040686 contained 9.2 (±0.9) ng/mL ML-I and 293 (±12) ng/mL ML-III.

For the study, two lots of mistletoe were manufactured, as the period of study enrollment spanned more than 2 years. The initial mistletoe lot was tested for ML-I and ML-III stability at the beginning of the study, and at 6, 15, and 18 months. ML-I and ML-III concentrations remained stable for 2 years.

### 2.2. Patient Recruitment and Screening Statistics

The protocol, informed consent, and patient recruitment materials were reviewed and approved by the National Cancer Institute's Institutional Review Board (IRB) on July 15, 2002 and by the National Naval Medical Center's IRB on December 12, 2002 (study number 02-AT-0260). A total of 704 persons expressed interest in this study and were contacted over this five year study. Forty-four persons (6%) were enrolled on-study after meeting all study eligibility criteria and signing written informed consent.

### 2.3. Study Eligibility Criteria

Patients with histologically confirmed treated or untreated, advanced pancreatic or non-small cell lung cancer (NSCLC), or recurrent metastatic colorectal or breast cancer were eligible for study participation. Additionally, study participants needed to be able and willing to administer daily subcutaneous injections of mistletoe by themselves or with assistance.

### 2.4. Study Design and Outcomes

The objective of this two stage, dose escalation phase I clinical trial was to observe the safety of the combination of gemcitabine and subcutaneously injected mistletoe extract in a population with advanced solid cancers and limited treatment options.

The study design, and rationale for this two agent, dose escalation paradigm, is published elsewhere [43]. In brief, in stage I, a fixed dose of gemcitabine (750 mg/m^2^) was administered intravenously on day 1 and day 8 of a 3-week cycle with an escalating mistletoe dose (1 mg, 5 mg, 10 mg, 20 mg, 50 mg, 100 mg, 200 mg, and 250 mg/day subcutaneously). As the manufacturer recommends mistletoe dosing from 50 to 200 mg, this dosing covered a range from 20% to 125% of the manufacturer recommended dose, which was considered a reasonable dosing range in a drug where there is precedence for clinical use and there are no prospective dosing data in combination with GEM. In stage II, a fixed mistletoe daily dose (as determined in stage I) was administered with gemcitabine in 20% dose increments per dose level (900, 1080, 1380, and 1560 mg/m^2^, resp., with the maximum dose being more than 50%, higher than the manufacturer recommended dose of 1000 mg/m^2^) [43]. This stage of the study examined whether participants' ability to tolerate gemcitabine would be differentially affected by concurrently administered mistletoe injections. Enrollment of 3 patients per dose level was planned. Grade 3 nonhematologic and grade 4 hematologic events were considered dose limiting toxicities (DLT). If three patients enrolled in a dose level successfully completed three cycles of the gemcitabine-mistletoe regimen with no DLT, then subsequently enrolled patients were assigned to the next higher dosage level. However, if 1 DLT occurred, an additional 1–3 patients were added to the cohort at that dose level for a maximum of 6 patients per dose level. The occurrence of 2 DLTs in one dosage level was considered to represent the maximum tolerated dose (MTD).

Primary study outcomes were the MTD and DLT of the combination regimen and the plasma gemcitabine pharmacokinetics alone and in combination with mistletoe extract. Secondary study outcomes were neutrophil count recovery, the stimulation of selected plasma cytokine levels (IL-6, IL-12, IFN*γ*, and TNF-*α*), the time to production and the circulating plasma concentrations of mistletoe lectin-1 (ML-1) and mistletoe lectin-3 (ML-3) antibodies, measured as IgG1-4 subclasses, and tumor response.

### 2.5. Participants and Data Collection Procedures

Enrolled participants were evaluated and treated in the hematology-oncology clinic at the National Naval Medical Center by the study investigators and NCI fellows. Prior to signing informed consent, the study investigators informed the participants about the purpose and methods of the study and explained where the study was in terms of stage and dose escalation. Once enrolled on-study, one of the study investigators instructed the study participant and family members on how to administer the daily subcutaneous mistletoe injections, with special attention to rotating the sites of injection and avoiding reinjection in the same area. Each participant (or family member) demonstrated successful subcutaneous administration of the mistletoe extract and was supplied with study supplies (i.e., sterile syringes, alcohol swabs, and sharps containers). Participants were informed that localized skin reactions, including discomfort at the injection site, redness, and itching, were commonly reported and were advised to inform the study staff if any skin reactions or other adverse events occurred.

Laboratory values were monitored twice a week, and clinical on-study evaluations were performed every cycle. CT scans were performed at baseline, and every 3 cycles. Adverse events were monitored weekly by the study investigators using ToxGrade, a software program designed for this study using the Common Terminology Criteria for Adverse Events (CTCAEv3) guidelines. Study data were tracked in a database monitored by the EMMES Corporation (Rockville, MD, USA). Independent study monitoring was provided by EMMES and KAI (both in Rockville, MD, USA).

### 2.6. Analytic Plan

The primary aim of this phase I study was to investigate the safety and toxicity of the mistletoe/GEM treatment regimen. As such, adverse events (any clinical event while on-study, considered related to mistletoe or gemcitabine based on published effects of the respective agents [44] rated as not related, possibly related, likely related, or definitely related), number of dose limiting toxicities, and clinical response (defined as progressive/stable disease or partial response at the time patients as assessed every 3 cycles and/or when patients were taken off of the study at disease progression, using RECIST criteria) are reported with corresponding descriptive statistics for the 44 study participants.

Secondary analyses included used Kaplan Meier [45] to assess time from study enrollment to death. Progression free survival and time to progression were initially considered but were difficult quantities to assess due to the lack of precise measurement of progression and assessment bias even when a rigorous definition is used (U.S. Food and Drug Administration CDER and CBER. Guidance for Industry Clinical Trial Endpoints for the Approval of Cancer Drugs and Biologics http://www.fda.gov/downloads/Drugs/GuidanceComplianceRegulatoryInformation/Guidances/ucm071590.pdf May 2007), and as such, time to disease progression analyses are not included in this paper.

An exploratory aim of the study was to examine potential trends of mistletoe and gemcitabine escalation on immune functioning. The *a priori* hypothesis was that ANC values would increase over the course of treatment; however, each group to be assessed had a small sample size. The Jonckheere-Terpstra trend test [46] was used to examine absolute neutrophil count (ANC) trends across time and across varying levels of gemcitabine and mistletoe treatments. This nonparametric statistical approach is similar to a Kruskal-Wallis test and has more power than the Kruskal-Wallis when there is *a priori* ordering of the populations from which the samples are drawn.

Pharmokinetics analyses used area under the curve analyses and plasma concentrations (CP, nmol/mL) from 20 minutes to 25 minutes following the infusion, comparing between cycle 1 (gemcitabine alone) and cycle 3 (gemcitabine plus mistletoe) using a Wilcoxon signed rank test.

## 3. Results

A total of 44 study participants were enrolled on this study; twenty patients were treated in stage I (mistletoe dose escalation phase) and 24 in stage II (gemcitabine dose escalation phase). The study population's demographic information is presented in Table 1(a). All patients had stage IV disease; the majority had received previous chemo-, hormonal, immunological, or radiation therapy, and 23% were chemotherapy-naïve. Patients' disease characteristics are listed in Table 1(b).

### 3.1. Adverse Events

A total of 706 discrete hematologic adverse events (AEs) were documented, occurring in 95% of study participants (Table 2(a)). The most common were low lymphocyte counts (for example, lymphopenia) (*n* = 200 events), anemia (*n* = 158), leukopenia (e.g., total WBC count) (*n* = 149), thrombocytopenia (*n* = 100), and neutropenia (e.g., low granulocyte or absolute neutrophil count) (*n* = 99). The majority (85%) of observed hematologic AEs were grade 1-2, 104 grade 3 (15%) and five grade 4 toxicities were observed. The grade 4 AEs included 1 neutropenia event (defined as an ANC < 500), 2 thrombocytopenia events (defined as platelet count < 25,000), and 2 lymphopenia events. A total of 570 nonhematologic AEs were recorded. The most common were hyperglycemia and hypoalbuminemia, followed by almost equal numbers of nausea and fatigue (Table 2(b)).

Nonneutropenic fever and flu-like syndrome, which have been previously described with mistletoe treatment and are also known AEs associated with gemcitabine, were observed in 24 of 44 (55%) patients. More patients experienced these symptoms in stage II of the study (15/24) than in stage I (9/20). Only one grade 3 febrile event was observed during stage II, all other events were grade 2 or less.

### 3.2. Mistletoe-Related Adverse Events

Mistletoe-related nonhematologic adverse events are represented in Table 3. A total of 112 adverse events were attributed to mistletoe treatment. The most common AEs attributed to mistletoe treatment were injection site reactions (42 events), localized induration (20 events), grade 1-2 nonneutropenic fever (22 events), and grade 1-2 flu-like symptoms (10 events). All of these AEs were expected as they had been documented as known mistletoe-related AEs in the Investigators' Brochure. Seventy-five events were grade 1, thirty-five were grade 2, and two events were grade 3. The two grade 3 events were cellulitis at the mistletoe injection site.

### 3.3. Gemcitabine-Related Adverse Events

A total of 473 hematologic AEs at least possibly related to gemcitabine were documented. Most commonly occurring number of events were leukopenia, thrombocytopenia, neutropenia, and anemia, which is consistent with the previously published data. Thirty patients developed a low WBC, and 28 patients developed thrombocytopenia. 30% of the low WBC events and close to 10% of the thrombocytopenic events were grade 3.

A total of 249 nonhematologic events were attributed at least possibly to gemcitabine. The most common were nausea (*n* = 47) and vomiting (*n* = 31), followed by liver enzyme elevation (elevated AST *n* = 25; elevated ALT *n* = 20), nonneutropenic fever (*n* = 21), and fatigue (*n* = 19). Thirteen grade 3 events were recorded, most commonly vomiting (*n* = 3) and fatigue (*n* = 2).

### 3.4. Maximum Tolerated Dose and Dose Limiting Toxicities

Five dose limiting toxicities (DLTs) were observed (Table 4). One study participant experienced grade 4 neutropenia at dose level 6 (mistletoe 250 mg/gemcitabine 900 mg/m^2^). An additional three participants enrolled onto this dose level did not subsequently experience a DLT. One study participant experienced grade 4 thrombocytopenia at dose level 7 (mistletoe 250 mg/gemcitabine 1180 mg/m^2^); three subsequent participants enrolled at this dose level did not experience a DLT. Three study participants experienced individual DLTs at dose level 9 (gemcitabine 1560 mg/m^2^ with 250 mg daily of mistletoe). These included grade 3 cellulitis at the mistletoe injection site, grade 4 acute renal failure, and grade 4 neutropenia. As per the protocol's study design, one dose level below the dose level at which 3 DLTs were reached was defined as the maximum tolerated dose. Thus, we achieved the MTD at dose level 8 (gemcitabine 1380 mg/m^2^ and mistletoe 250 mg).

### 3.5. Pharmacokinetics of Gemcitabine

Plasma concentrations of gemcitabine from patients treated during stage I were measured in nmol/mL. Fifteen of 20 patients treated in stage I had plasma samples obtained for analysis. Twelve patients had paired samples obtained during cycle 1 (without mistletoe) and cycle 3 (with mistletoe).

The addition of mistletoe did not affect gemcitabine pharmacokinetics as measured during cycle 3 (cycle 3, day 8 of gemcitabine/mistletoe combination) compared to cycle 1 of treatment (gemcitabine alone on day 1 of treatment before mistletoe was added on day 8, *P* values ranging from 0.47 to 0.97; Table 5).

### 3.6. Best Clinical Response to Treatment

**Figure 1:**
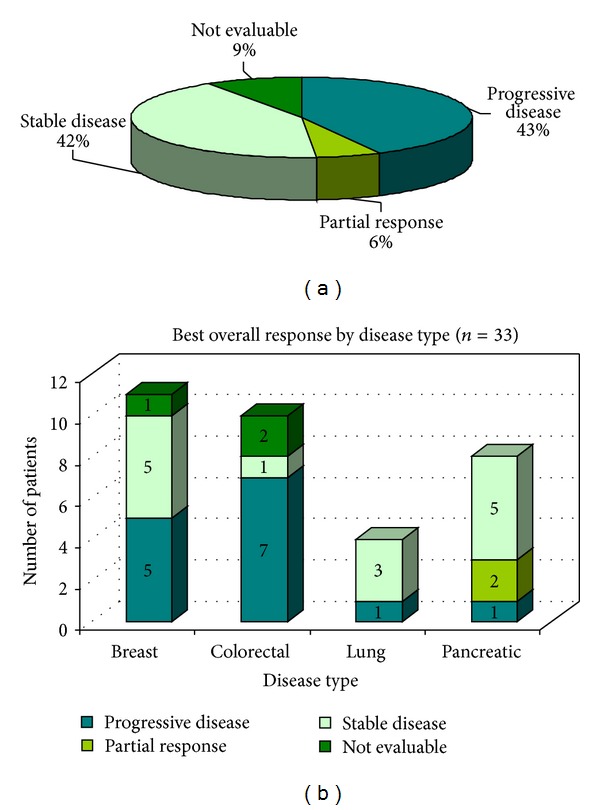
(a) Best clinical response and (b) best overall response by diagnosis.

Figure 1(a) shows the best overall response. Of the 44 enrolled study patients, 33, completed at least 3 cycles of therapy. Of these 33, six percent (*n* = 2) had a partial response, 42% (*n* = 14) had stable disease, and 43% (*n* = 14) progressed on treatment. Nine percent (*n* = 3) were not evaluable for response.

### 3.7. Best Clinical Response according to Diagnosis

Both partial responses were observed in patients with pancreatic cancer. Three of 4 evaluable patients with NSCLC had stable disease, and 5/11 patients with breast cancer had stable disease (Figure 1(b)). Only 1 out of 8 patients with colorectal cancer had stable disease.

### 3.8. Survival Analyses

**Figure 2:**
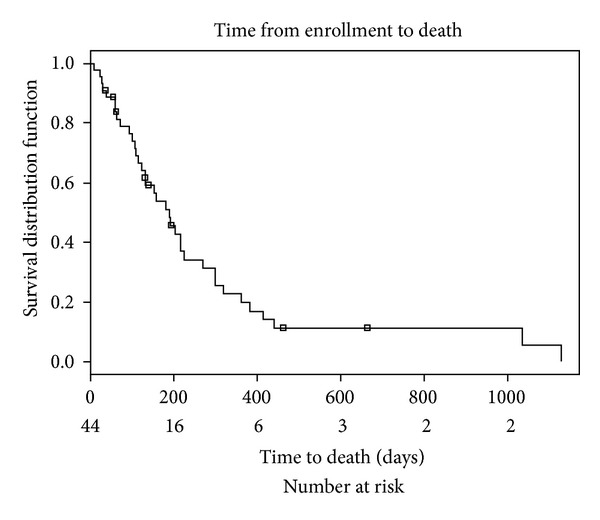
Time from enrollment to death.

Of the 44 study participants, three participants died on study, 10 participants requested to terminate the study, 23 participants progressed while on study, one terminated the study due to a dose limiting toxicity, 6 left due to complicating disease issues which may be tied to progression, and one voluntarily withdrew. An attempt was made to follow study subjects once they terminated study treatment until death. At the study's last attempt to contact former participants, three were still alive and five others were lost to followup. A Kaplan Meier curve was used to illustrate time to death in Figure 2. The median time to death of any cause was approximately 200 days.

### 3.9. Jonckheere-Terpstra Trend Test Results for ANC Values across Increasing Dose Levels

We prospectively followed ANC nadir and ANC maximum as one of the study outcomes, hypothesizing that the ANC may be influenced by mistletoe exposure. ANC values showed a trend for increase between baseline and cycle 2 in stage I (*P* = 0.06). When ANC maximum was measured, there was a significant trend (*P* = 0.034) for the maximum ANC level achieved in stage II during cycle 1. However, if patients were eliminated based on dexamethasone exposure, the trend for stage I diminished (*P* = 0.092) but was maintained for cycle 1 ANC maximum during stage II (*P* = 0.017).

### 3.10. Development of Mistletoe Lectin 3 IgG Antibodies and Cytokine Release

Helixor A extract is low in ML-1 and high in ML-3 content. Therefore, ML-3 content was followed. All study patients eventually developed mistletoe lectin 3 IgG antibodies. The formation of antibodies was higher at increasing doses of mistletoe.

For stage II, when all patients were exposed to the same mistletoe regimen with increasing doses of gemcitabine, only IgG3 antibody levels increased with increasing doses of gemcitabine (data not shown). Cytokines were minimally affected by this combination regimen.

## 4. Discussion

To our knowledge, this is the first reported dose escalation study of a whole mistletoe extract combined with single agent gemcitabine in patients with advanced solid cancers. As per the manufacturer's information [44], nonfebrile neutropenia as a function of dose in gemcitabine was observed in 63% (19% grade 3, 6% grade 4). Thrombocytopenia occurred in 24% (4% grade 3, 1% grade 4). Gemzar as a single agent was administered at doses between 800 mg/m^2^ and 1250 mg/m^2^ over 30 minutes intravenously, once weekly, in 979 patients with a variety of malignancies.

We observed 30% grade 3 neutropenia and 10% grade 3 thrombocytopenia, while single agent gemcitabine testing has resulted in 19% grade 3 nonfebrile neutropenia and 4% grade 3 thrombocytopenia [44]. Thirty-seven percent of patients experienced nonneutropenic fever, while single agent gemcitabine was associated with 41% fever. There was no documented incidence of febrile neutropenia for the combination regimen. Flu-like symptoms occurred in 18% of patients.

The hematologic toxicity profile of the mistletoe/gemcitabine combination and febrile reactions in this study were similar to single agent gemcitabine [44]. The addition of mistletoe did not exacerbate hematologic gemcitabine toxicity. Interestingly, there was a trend (*P* = 0.06) towards increased ANC nadir during the first 3 weeks of initiation of mistletoe and of ANC maximum during the first 6 weeks as a function of mistletoe dose (*P* = 0.034). Others have claimed that mistletoe may boost chemotherapy tolerance, but published data on dosing and mistletoe schedule are lacking, while these were collected in detail in this study.

Flu-like symptoms may be more common when mistletoe is added to gemcitabine. We observed febrile and flu-like reactions attributable to mistletoe across the entire mistletoe dosing spectrum that did not seem to be dose dependent.

The addition of mistletoe did not affect the pharmacokinetics of gemcitabine at any of the mistletoe dose levels tested, suggesting that mistletoe can be added to gemcitabine without concern about adversely affecting gemcitabine's pharmacokinetic profile. The MTD for the gemcitabine/mistletoe combination in this study was gemcitabine 1380 mg/m^2^ given weekly on day one and eight of a three-week cycle with mistletoe 250 mg s.c. daily. As per the manufacturer recommendations, gemcitabine is commonly dosed at 1000 mg i.v. weekly for three weeks on a 28-day cycle. In our study a higher dose was tolerated.

Stimulatory effects of mistletoe on neutrophils and lymphocytes have been reported *in vitro *as well as in patients. We observed a mistletoe dose-dependent trend towards increased absolute neutrophil count ANC nadir during cycle 1 and ANC maximum during cycle 2. None of the study patients developed febrile neutropenia even at the highest gemcitabine dose of 1650 mg/m^2^. As this study employed a dose escalation scheme in a diverse group of patients with advanced cancer, many of whom were heavily pretreated, this observation would have to be verified and confirmed in a setting of increased homogeneity of patient population and treatment regimen with a larger sample size.

There is a sizable body of literature on the effects of mistletoe on cytokine production [21]. We selected testing for IL-6, IL-12, IFN gamma, and TNF alpha based on their previously described role in tumor development and proliferation as well as existing publications of possible effects of mistletoe on the production of these cytokines. The production of these cytokines in patients with cancer however has not been studied in detail when chemotherapy was combined with mistletoe. We did not detect any consistent pattern of increased or decreased production of any of the cytokines tested.

Mistletoe lectin (ML)-3 antibody formation of the IgG type was detected in all patients by cycle 3 of therapy or 9 weeks and thus was independent of the actual mistletoe dose administered. The physiologic effect of the formation of ML antibodies is not well understood. While we did not compare participants' injections site reactions to this immunological data, we did observe injection site reactions early in treatment of all study participants. Others have reported local reactions in 87% [31]. In most studies, mistletoe is injected three times per week, while our patients injected mistletoe daily. It is thus not surprising that our study would find a higher rate of local injection site reactions. Skin reactions decreased over the course of therapy. This phenomenon may have resulted from the increasing formation of ML antibodies over time mitigating the mistletoe related injection site reactions. Febrile reactions occurred in more than one-third of the patients. It is not clear from our data that there was any relationship between the appearance of ML antibodies and febrile reactions or other toxicities. The study was not designed to yield reliable data on clinical response to the study regimen. Therefore, we are unable to determine associations between clinical response and the formation of ML antibodies or febrile reactions. Future studies may add the understanding of the physiological reactions to mistletoe therapy by connecting immunological data to changes in symptom presentation.

The finding of a partial response rate of 6% is comparable to what would be expected from single agent gemcitabine in this population of patients with advanced, mostly heavily pretreated carcinomas. The median survival from study enrollment of about 200 days is within the range of what would be expected from single agent gemcitabine. Compliance with the mistletoe regimen was high, and no episode of febrile neutropenia was observed in any of the 44 patients. The lack of episodes of febrile neutropenia in a set of heavily pretreated patients of whom almost 50% received gemcitabine doses of 1100 mg/m^2^ or higher is noteworthy, but would have to be confirmed in a larger, more homogenous cancer population.

The above results should be interpreted in light of several study limitations. First, the study sample included 4 different types of solid tumors, each of which may respond differently to GEM or mistletoe/GEM therapy. As such, the results presented herein may not extend to more homogenous groups of cancer patients. Second, the overall sample size was small (*n* = 44). As a result, the study may have been underpowered to detect significant trends for the study outcomes. Despite the majority of study outcomes remaining descriptive in nature, the statistical tests applied (e.g., survival analyses and Jonckheere-Terpstra trend test) should be interpreted in light of the small sample sizes used in each respective test. The overall small sample size precluded subgroup analyses (e.g., treatment naïve participants' response), which may be a worthwhile area for future studies to pursue. Finally, the study was not designed to examine the effectiveness of a mistletoe/GEM combination. Future work should apply double blind, randomized controlled study designs to examine.

## 5. Conclusion

The combination of mistletoe and gemcitabine was well tolerated and treatment compliance was high. The MTD was gemcitabine 1380 mg/m^2^ weekly on day one and eight of a 3-week cycle combined with mistletoe 250 mg daily. Gemcitabine pharmacokinetics were not affected by mistletoe. The lack of febrile neutropenia even at higher gemcitabine doses is noteworthy. The formation of ML antibodies is common. A consistent effect of the study regimen on the serum levels of selected cytokines could not be demonstrated. Clinical response of the combination appeared to be similar to single agent gemcitabine reported previously.

## Figures and Tables

**Table:** (a)

	Stage I (*n*)	Stage II (*n*)	Total (*n*)	Total (%)
Number enrolled	20	24	44	—
Age (years)				
Mean	55.0	55.1	55.1	—
Range	29–81	29–76	29–81	—
Gender				
Male	10	13	23	52%
Female	10	11	21	48%
Race				
White	17	19	36	82%
Black	2	2	4	9%
Asian	0	3	3	7%
Ethnicity				
Not Hispanic	17	24	41	93%
Hispanic	3	0	3	7%
Cancer diagnosis				
Colorectal	4	13	17	39%
Breast	6	6	12	27%
Pancreatic	6	4	10	23%
Lung	4	1	5	11%
Disease stage				
IV	20	24	44	100%

**Table:** (b)

Disease type	No prior treatment	Chemotherapy only	Radiation only	Chemotherapy and radiation	Chemotherapy and surgery	Chemotherapy, surgery, and radiation	Total
Colorectal	0	4	0	1	8	4	17
Breast	0	1	0	0	1	10	12
Pancreatic	9	0	0	1	0	0	10
Lung	1	1	1	2	0	0	5

Total (*n*/%)	10 (22.7%)	6 (13.6%)	1 (2.3%)	4 (9.1%)	9 (20.5%)	14 (31.8%)	44

*No study participants were treated solely with surgery or with surgery plus radiation only.

**Table:** (a)

Hematologic adverse events	Number of events (possibly multiple events from same participant)					Number of participants experiencing (multiple) events	Number of participants (with most severe event if there are multiple)			
	Grade 1	Grade 2	Grade 3	Grade 4	Total		Grade 1	Grade 2	Grade 3	Grade 4
Lymphopenia	42	105	51	2	200	34		15	17	2
Anemia	85	61	12		158	41	13	22	6	
Leukopenia (Total WBC)	83	50	16		149	30	8	12	10	
Thrombocytopenia	78	13	7	2	100	29	20	4	3	2
Neutropenia (ANC/AGC)	41	39	18	1	99	26	4	11	10	1
Total hematologic AEs	329	268	104	5	706					

**Table:** (b)

Nonhematologic adverse events	Number of events (possibly multiple events from same participant)					Number of participants experiencing (multiple) events	Number of participants (with most severe event if there are multiple)			
	Grade 1	Grade 2	Grade 3	Grade 4	Total		Grade 1	Grade 2	Grade 3	Grade 4
Hyperglycemia	81	35	8		124	34	17	12	5	
Hypoalbuminemia	53	38	2		93	29	11	16	2	
Hypocalcemia	59	19	1		79	33	20	12	1	
Hyponatremia	50	0	9	1	60	29	22	0	6	1
Elevated AST, SGOT	42	13	2		57	30	20	8	2	
Elevated ALP, ALKP	29	22	5		56	25	10	10	5	
Nausea	27	23	1		51	24	9	14	1	
Fatigue	20	25	5		50	29	7	17	5	
Total: most commonly occurring nonheme AEs	361	175	33	1	570		116	89	27	1
Total: nonheme AEs (overall)	751	398	85	6	1243					

**Table 3:** Nonhematologic CTCAE adverse events at least possibly related to mistletoe (*n* = 44).

Nonhematologic adverse events	Number of events (possibly multiple same events from participant)				Number of participants experiencing (multiple) events	Number of participants (with most severe event if there are multiple)		
	Grade 1	Grade 2	Grade 3	Total		Grade 1	Grade 2	Grade 3
Injection site reaction	30	12		42	26	14	12	
Fever (in the absence of neutropenia)	18	4		22	14	11	3	
Induration/fibrosis skin and subcutaneous tissue	13	7		20	15	8	7	
Flu-like syndrome	6	4		10	9	5	4	
Pruritus	3	1		4	3	2	1	
Cellulitis (with normal ANC or grade 1 or 2 ANC)		1	1	2	2		1	1
Allergic reaction/hypersensitivity		1		1	1		1	
Dermatology skin reaction-NOS		1		1	1		1	
Cellulitis with unknown ANC			1	1	1			1
Lymphatics-NOS	1			1	1	1		
Lymphedema		1		1	1		1	
Myalgia NOS	1			1	1	1		
Pain-joint		1		1	1		1	
Pain-skin	1			1	1	1		
Phlebitis		1		1	1		1	
Rash: erythema multiforme		1		1	1		1	
Rigors/chills	1			1	1	1		
Total nonheme AEs	75	35	2	112				

NOS: not otherwise specified.

**Table 4:** Dose limiting toxicities by dose level.

Stage I (fixed GEM dose of 750 mg/m^2^)			Stage II (fixed mistletoe dose, established in stage 1)			
Level	*n*	DLT	Level/dosage	*n*	DLT	Action taken per protocol
(1) (escalating daily mistletoe injections, reaching: 20 mg/day)	3	None	(6) (250 mg/day mistletoe; **900** mg/m^2^ GEM on day 1/8 of 3-week cycle)	7	Grade 4 neutropenia	Dose reduced; enrolled 3 more patients at this dose level
(2) (escalating daily mistletoe injections, reaching: 50 mg/day)	3	None	(7) (250 mg/day mistletoe; **1080** mg/m^2^ GEM on day 1/8 of 3-week cycle)	7	Grade 4 thrombocytopenia	Dose reduced; enrolled 3 more patients at this dose level
(3) (escalating daily mistletoe injections, reaching: 100 mg/day)	3	None	(8)* (250 mg/day mistletoe; **1380** mg/m^2^ GEM on day 1/8 of 3-week cycle)	6	None	N/A
(4) (escalating daily mistletoe injections, reaching: 200 mg/day)	6	None	(9) (250 mg/day mistletoe; **1560** mg/m^2^ GEM on day 1/8 of 3-week cycle)	4	Grade 3 cellulitis^a^; grade 4 acute renal failure^b^; grade 4: thrombocytopenia^c^	^ a^Mistletoe therapy withheld; patient rechallenged and developed hypersensitivity reaction. Mistletoe discontinued; ^b^Pt. treated for renal failure and subsequently withdrawn from study; ^c^Pt. hospitalized d/t other AE; *Maximum tolerated dose reached per protocol*
(5) (escalating daily mistletoe injections, reaching: 250 mg/day)	5	None				

*Per study protocol, this level represents the maximum tolerated dose, as 3 DLT's were observed in the subsequent dose level.

**Table 5:** Pharmacokinetics of gemcitabine (cycle 1) and gemcitabine plus mistletoe (cycle 3).

	Cycle 1 Median (25th%, 75th%)	Cycle 3 Median (25th%, 75th%)	*P* value Signed rank test
Gemcitabine AUC (min ∗ nmol/mL)	664 (514, 870)	670 (625, 851)	0.97
Gemcitabine Average Cp (nmol/mL)	47.7 (42.7, 64.6)	49.7 (45.7, 53.8)	0.85
